# Supplementary figures and images for: Codon Bias Patterns of E. coli’s Interacting Proteins
Source: PLoS One. 2015 Nov 13;10(11):e0142127. doi: 10.1371/journal.pone.0142127 (PMC4643964; doi:10.1371/journal.pone.0142127)

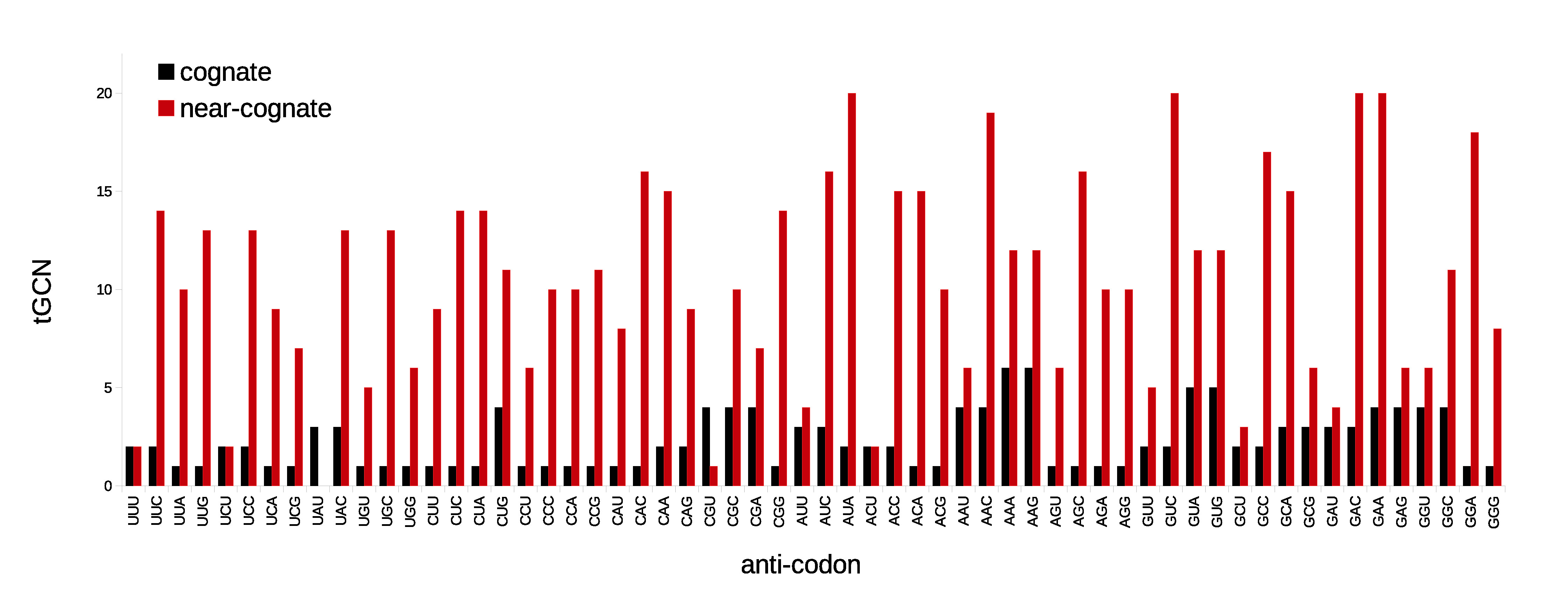

Supplement: S1 Fig — Data taken from [17]. (TIFF) [file pone.0142127.s001.tiff]

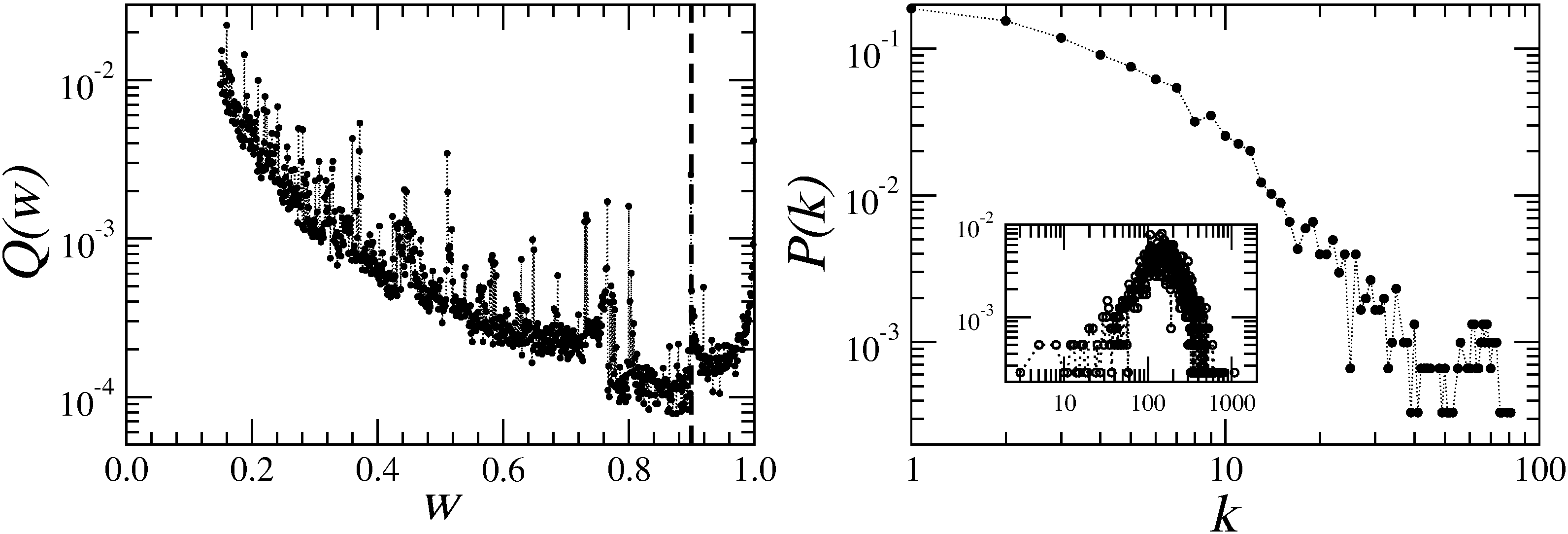

Supplement: S2 Fig — Left plot: Distribution of confidence levels Q(w), with the vertical line indicating the cut-off we use to separate true from false positives. Right plot: Distribution of degrees P(k) when Θ = 0.9, with the insets showing the same distribution for the original network (Θ = 0). (TIFF) [file pone.0142127.s002.tiff]

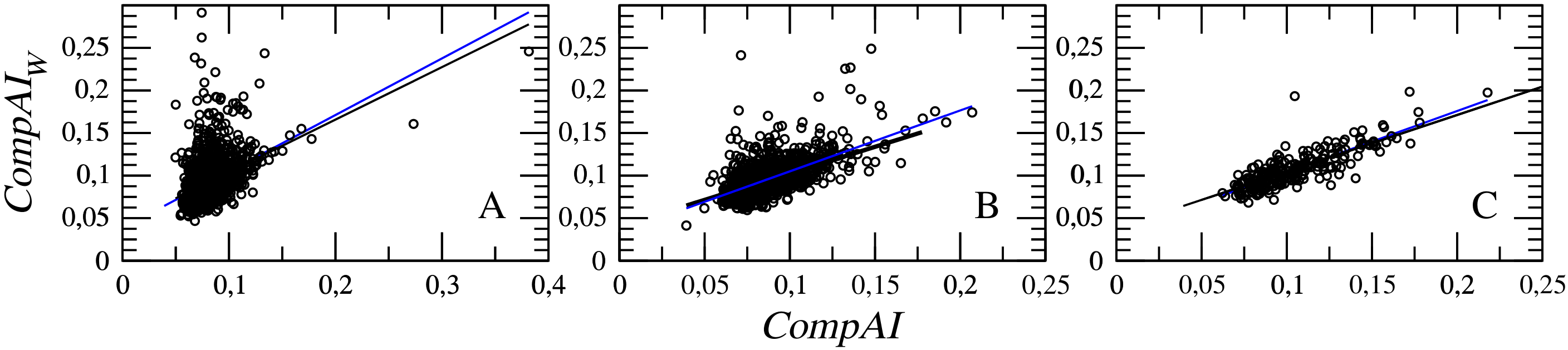

Supplement: S3 Fig — Codon bias values are plotted separately for each of the E.Coli gene groups A, B and C. The black solid line identifies the linear regression over the whole genome (with Pearson’s correlation coefficient c = 0.58). The blue solid lines instead represent linear regression on individual groups, with correlation coefficients c = 0.43 for group A, c = 0.62 for group B and c = 0.83 for group C. (TIFF) [file pone.0142127.s003.tiff]
