# Supplementary material for: Codon Bias Patterns of E. coli’s Interacting Proteins
Source: PLoS One. 2015 Nov 13;10(11):e0142127. doi: 10.1371/journal.pone.0142127 (PMC4643964; doi:10.1371/journal.pone.0142127)
Supplement: S1 File — (PDF) [file pone.0142127.s004.pdf]

Codon Bias Patterns of *E.coli*'s Interacting Proteins

Maddalena Dilucca[1,*], Giulio Cimini[2], Andrea Semmoloni[1], Antonio Deiana[1], Andrea Giansanti[1,3]

1 Dipartimento di Fisica, Sapienza University of Rome, Rome, Italy
2 Istituto dei Sistemi Complessi (ISC-CNR) UoS Sapienza University of Rome, Rome, Italy
3 INFN Roma1 unit, Rome, Italy

*** Maddalena.Dilucca@roma1.infn.it**

Supporting Information

Here we define and explain the rationale behind the various codon bias indices that have been proposed in the literature.

Codon Adaptation Index (CAI) [1]. The pattern of codon usage is ruled by two simultaneous effects [2]: translational selection towards optimal codons for each amino acid, and genetic drift that allows the persistence of non-optimal codons. It is natural to assume that selection is stronger for codons of highly expressed genes, which thus feature a more pronounced bias in the use of codons. The principle behind CAI is that codon usage in highly expressed genes can reveal the optimal (*i.e.*, most efficient for translation) codons for each amino acid. Hence, CAI builds on a reference set of highly expressed genes to assess, for each codon i, the relative synonymous codon usages ($RSCU_i$) and the relative codon adaptiveness (w_i):

$$RSCU_i = \frac{X_i}{\frac{1}{n_i}\sum_{j=1}^{n_i} X_j}; \qquad w_i = \frac{RSCU_i}{\max_{j=1,\ldots,n_i}\{RSCU_j\}}; \tag{1}$$

In the $RSCU_i$, X_i is the number of occurrences of codon i in the genome, and the sum in the denominator runs over the n_i synonyms of i; $RSCU$s thus measure codon usage bias within a family of synonymous codons. w_i is then defined as the usage frequency of codon i compared to that of the optimal codon for the same amino acid encoded by i—*i.e.*, the one which is mostly used in a reference set of highly expressed genes.

The CAI for a given gene g is calculated as the geometric mean of the usage frequencies of codons in that gene, normalized to the maximum CAI value possible for a gene with the same amino acid composition:

$$CAI_g = \left(\prod_{i=1}^{l_g} w_i\right)^{1/l_g}, \tag{2}$$

where the product runs over the l_g codons belonging to that gene (except the stop codon). The critical aspect in the definition of CAI is that it requires to define *a priori* reference set of highly expressed genes that is specific for a given organism. CAI is then not always transferable; yet, since it is tuned on highly expressed genes, it is generally

well correlated with gene expression levels in genomes for which reference gene sets are available. In this work, CAI for E.coli genes was computed using the DAMBE 5.0 package [3]

tRNA Adaptation Index (tAI) [4]. The speed of protein synthesis is bound to the waiting time for the correct tRNA to enter the ribosomal A site [5], and thus depends on tRNA concentrations [6] (and, indirectly, on gene copy numbers). The consequent adaptation of codon usage to tRNA availability [7,8] is at the basis of tAI, which follows the same mathematical model of CAI—defining for each codon i its absolute (W_i) and relative (w_i) adaptiveness value:

$$W_i = \sum_{j=1}^{m_i} (1 - s_{ij})\, \mathrm{tGCN}_{ij}; \qquad w_i = \begin{cases} W_i/W_{max} & \text{if } W_i \neq 0 \\ w_{mean} & \text{otherwise} \end{cases}; \tag{3}$$

where m_i is the number of isoacceptor tRNAs that recognize codon i (*i.e.*, tRNAs that carry the same amino acid that is encoded by i and that make either WC or wobble pairing with it), tGCN_{ij} is the gene copy number of the j-th tRNA that recognizes the i-th codon, s_{ij} is a selective constraint on the efficiency of the codon-anticodon coupling, W_{max} is the maximum W_i value and w_{mean} is the geometric mean of all w_i with $W_i \neq 0$.

The tAI of gene g is eventually defined as the geometric mean of the relative adaptiveness values of its codons, thus estimating the amount of adaptation of gene g to its genomic tRNA pool:

$$tAI_g = \left(\prod_{i=1}^{l_g} w_i \right)^{1/l_g}. \tag{4}$$

The critical issue for tAI is the selection of a meaningful set of s_{ij} values, *i.e.*, weights that represent the efficiency of the interactions between codons and tRNAs. Assuming that tRNA usage is maximal for highly expressed genes, these values are chosen in order to optimize the correlation of tAI values with expression levels—exactly as CAI. Besides, while the efficiencies of the different codon-tRNA interactions are expected to vary among different organisms, s_{ij} values are based on the gene expression in *Saccharomyces cerevisiae* [4]—thus lacking universality [9]. In this work we have evaluated tAI values of E.coli genes using the CodonR package [10].

Effective Number of Codons (Nc) [11]. Nc is a measure that quantifies the departure of a gene from the random usage of synonymous codons. Given a sequence of interest, the computation of Nc [12] starts from the quantity—defined for each family α of synonymous codons:

$$F_{CF_\alpha} = \sum_{k=1}^{m_\alpha} \left(\frac{n_{k_\alpha}}{n_\alpha} \right)^2 \tag{5}$$

where m_α is the number of codons in α (each appearing $n_{1_\alpha}, n_{2_\alpha}, \ldots, n_{m_\alpha}$ times in the sequence) and $n_\alpha = \sum_{k=1}^{m_\alpha} n_{k_\alpha}$. Nc then weights these quantities in order to measure amount of entropy in the codon usage of the sequence:

$$Nc = N_S + \frac{K_2 \sum_{\alpha=1}^{K_2} n_\alpha}{\sum_{\alpha=1}^{K_2} (n_\alpha F_{CF_\alpha})} + \frac{K_3 \sum_{\alpha=1}^{K_3} n_\alpha}{\sum_{\alpha=1}^{K_3} (n_\alpha F_{CF_\alpha})} + \frac{K_4 \sum_{\alpha=1}^{K_4} n_\alpha}{\sum_{\alpha=1}^{K_4} (n_\alpha F_{CF_\alpha})} \tag{6}$$

where N_S is the number of families with one codon only and K_m is the number of families with degeneration m (families with degeneration 6 are divided into two families

of degeneration 2 and 4, as they often are subject to different selective forces). Note that Nc reaches is maximal value (61) when all codons are used equally and its minimal value (23) when only one codon is used per amino acid (extreme bias). Differently from both CAI and tAI, Nc is a more immediate measure of codon usage that does not require any *a priori* information nor makes any biological hypothesis (which constitute its weakness and, and the same time, its strength). Yet, since the effect of selection is a reduction of entropy for codon usage in a sequence, Nc provides a reliable measure for this effect. In this paper we have obtained Nc values through DAMBE 5.0 [3].

References

1. Sharp PM, Li WH. The codon Adaptation Index—a measure of directional synonymous codon usage bias, and its potential applications. Nucleic Acids Res. 1987;15(3):1281–1295.
2. Bulmer M. The selection-mutation-drift theory of synonymous codon usage. Genetics. 1991;129(3):897–907.
3. Xia X. DAMBE5: a comprehensive software package for data analysis in molecular biology and evolution. Mol Biol Evol. 2013;30(7):1720–8.
4. dos Reis M, Savva R, Wernisch L. Solving the riddle of codon usage preferences: a test for translational selection. Nucleic Acids Res. 2004;32(17):5036–5044.
5. Varenne S, Buc J, Lloubes R, Lazdunski C. Translation is a non-uniform process. Effect of tRNA availability on the rate of elongation of nascent polypeptide chains. J Mol Biol. 1984;180(3):549–576.
6. Sørensen MA, Kurland CG, Pedersen S. Codon usage determines translation rate in Escherichia coli. J Mol Biol. 1989;207(2):365–377.
7. Ikemura T. Correlation between the abundance of Escherichia coli transfer RNAs and the occurrence of the respective codons in its protein genes: a proposal for a synonymous codon choice that is optimal for the E. coli translational system. J Mol Biol. 1981;151(3):389–409.
8. Ikemura T. Codon usage and tRNA content in unicellular and multicellular organisms. Mol Biol Evol. 1985;2(1):13–34.
9. Sabi R, Tuller T. Modelling the efficiency of codon-tRNA interactions based on codon usage bias. DNA Res. 2014;21(5):511–26.
10. `http://people.cryst.bbk.ac.uk/~fdosr01/tAI/index.html`
11. Wright F. The "effective number of codons" used in a gene. Gene. 1990;87(1):23–29.
12. Sun X, Yang Q, Xia X. An improved implementation of Effective Number of Codons (Nc). Mol Biol Evol. 2012;30(1):191–196.
